# Comparative analysis of vibration-controlled transient elastography and EUS-shear wave elastography for liver stiffness measurement in cirrhosis

**DOI:** 10.1097/eus.0000000000000114

**Published:** 2025-05-05

**Authors:** Raquel del Valle, Domenica Cunto, Miguel Puga-Tejada, Maria Egas-Izquierdo, Martha Arevalo-Mora, Roberto Oleas, Juan Alcivar-Vasquez, Fernanda Dal Bello, Hannah Pitanga-Lukashok, Jorge Baquerizo-Burgos, Carlos Robles-Medranda

**Affiliations:** 1 Instituto Ecuatoriano de Enfermedades Digestivas (IECED), Guayaquil, Ecuador; 2 Internal Medicine, Larkin Community Hospital, South Miami, FL, USA; 3 John H. Stroger, Jr. Hospital of Cook County, Chicago, IL, USA.

**Keywords:** Liver cirrhosis, Fibrosis, EUS, Elastography

## Abstract

**Background and Objectives:**

Chronic liver inflammation leads to fibrosis and cirrhosis. To avoid portal hypertension-related complications, fibrosis' early detection is imperative. Biopsy remains the gold standard, but magnetic resonance elastography (MRE) and EUS-guided elastography are noninvasive procedures currently used for liver stiffness measurement (LSM). Two-dimensional EUS-guided shear-wave elastography (EUS-SWE) represents a more-every-day used technique.

The aim of this study is to correlate LSM determined by vibration-controlled transient elastography (VCTE) and EUS-SWE and determine the measurements' accuracy in diagnosing cirrhosis.

**Methods:**

A single-center, nested case-control study was performed between March 2020 and November 2021. Patients were classified into 2 study groups: the cirrhosis group and the control group. Patients from both groups underwent VCTE and EUS-SWE for LSM. A *P* value < 0.05 was considered statistically significant.

**Results:**

Of the 59 participants included (mean age 63.5 years; 71.1% female), 29 had cirrhosis (49.15%) and 30 were controls (50.84%). In cirrhosis patients, liver fibrosis (F) was staged as F3–4 by VCTE in 82.8%, with a median LSM of 17.8 kPa; through EUS-SWE, 27 kPa in the right hepatic lobe (RHL) and 25 kPa in the left hepatic lobe (LHL). Controls fibrosis was staged as F0–2 by VCTE in 30/30 (100%), with a median LSM of 4.6 kPa (*P* < 0.001); through EUS-SWE, 5.6 kPa in the RHL (*P* < 0.001) and 6.5 kPa in the LHL (*P* < 0.001). The observed agreement was 91.5% for VCTE, 93.2% for RHL-EUS-SWE, and 96.6% for LHL-EUS-SWE. The AUROCs for EUS-SWE and VCTE were over 0.95.

**Conclusions:**

VCTE and EUS-SWE are comparable techniques for diagnosing cirrhosis; however, EUS-SWE had a higher agreement than VCTE, especially in LHL assessment.

## INTRODUCTION

Chronic inflammation of the liver, regardless of the cause, can lead to liver fibrosis and the subsequent development of cirrhosis. Both diseases are progressive and a major health concern, causing approximately 1.5 million deaths per year worldwide, and together have become the fifth leading cause of death for persons aged 40–59 years.^[1,2]^ Progressive fibrosis can lead to complications, such as portal hypertension or hepatocellular carcinoma.^[3]^ Therefore, it is necessary to detect fibrosis at early stages and accurately assess disease severity to avoid further complications.^[1,2]^

Liver biopsy remains the gold standard for staging liver fibrosis. It must be obtained in cases where the diagnosis, prognosis, or management of cirrhosis is uncertain. Nonetheless, liver biopsy is invasive and has several limitations, such as sampling error; adverse events such as pain, bleeding, or perforation, and the associated increase in healthcare-related costs.^[1,2,4]^ Moreover, interobserver disagreement associated with grading and staging is another drawback. For instance, in the early stages of nonalcoholic fatty liver disease (NAFLD), the concordance of staging is 26.7% in the steatosis stage, 62.7% in the inflammatory phase, 51.3% in ballooning, 48.7% in fibrosis, and 50.7% in steatohepatitis.^[5]^ Thus, liver biopsies at multiple sites are required to account for the heterogeneous distribution of fibrosis.^[3]^

The limitations of liver biopsy have motivated the development of minimally invasive tests and diagnostic devices for the assessment of liver fibrosis. Currently utilized noninvasive techniques include magnetic resonance elastography (MRE) and ultrasound (US)-based elastography,^[6]^ but the latter is preferred due to lower cost and easier instrument manipulation. To date, 5 types of US-based elastography are available, broadly grouped as imaging-based methods (strain elastography and acoustic radiation force impulse) and shear wave (SW) imaging-based methods (point shear wave elastography [pSWE], 1-dimensional vibration-controlled transient elastography [VCTE], and 2-dimensional shear wave elastography [2D-SWE]).^[6]^

VCTE, an innovative US-based elastography technique, is currently the most commonly used method for liver fibrosis evaluation. However, it has limited utility in patients with narrow rib spaces or the presence of ascites, and an additional XL probe is necessary to assess obese patients.^[1,3,7]^ EUS-guided SWE (EUS-SWE) provides a real-time quantitative mapping of liver stiffness, with a larger area of liver sampling and faster analysis to display color-mapped shear wave values compared to ultrasound.^[1,6,8]^ This study aimed to compare the diagnostic accuracy of VCTE and EUS-SWE for the prediction of liver cirrhosis and correlate LSM with both techniques.

## MATERIALS AND METHODS

### Study design

#### Study type

The following was an independent, single-center, cross-section diagnostic trial. Two study groups were included in this study: cirrhosis patients and controls. The study design fulfilled the Standards for Reporting Diagnostic Accuracy (STARD) 2015 statement.

#### Setting

Participants in both study groups underwent EUS-SWE evaluation at the *Instituto Ecuatoriano de Enfermedades Digestivas (IECED),* Guayaquil, Ecuador, between March 2020 and November 2021. The local institutional review board (IRB) approved the study, which was conducted following the Declaration of Helsinki.

### Population and sample

#### Cirrhosis group

Patients in this group were referred to the Hepatology Clinic with a recent diagnosis of liver cirrhosis based on clinical presentation, abdominal ultrasonography, and laboratory findings. The liver cirrhosis diagnosis must be established within 30 days before trial enrollment. Patients were invited to undergo VCTE and EUS-SWE for liver evaluation, followed by a confirmatory EUS-guided 2-lobe liver biopsy. Patients with a Child-Pugh C score or MELD score >19, hepatocellular carcinoma suspicion, history of uncontrolled coagulopathy, or any other contraindications for EUS-guided liver biopsy, as well as pregnant or nursing patients and those who did not agree to participate, were excluded from the study.

#### Control group

Participants in this group were enrolled from a clinical database of patients with subepithelial lesions that were followed up and evaluated by EUS, or patients with chronic dyspepsia who required EUS for clinical suspicion of subepithelial lesions. Patients were invited to undergo an EUS-SWE evaluation of liver stiffness in addition to the standard EUS evaluation for subepithelial lesion follow-up. Patients with a history of alcohol use or hepatotoxic medication consumption were excluded from the study. Patients who did not agree to participate and those who were either pregnant or nursing were excluded from the study. However, patients with overweight, obesity, non–hepatic-related low platelet counts, elevated aspartate transaminase (AST) or alanine aminotransferase (ALT) levels, or VCTE-estimated steatohepatitis were not excluded to account for the presence of these conditions in the general population.

### Procedure and technique

#### Initial clinical assessment

A general practitioner and clinical research fellow (D.C.) was responsible for patient selection and clinical assessment before the VCTE and EUS-SWE evaluations. The following data were recorded: age, sex, and body mass index (BMI), cirrhosis-related data if indicated (cause of cirrhosis, alcohol intake, Child-Pugh score, and MELD score), and serum platelet, AST, and ALT levels. Patients were blindly and consecutively addressed to VCTE and EUS-SWE by D.C.

#### Vibration-controlled transient elastography

VCTE was performed by H.P.-L., who was blinded to all clinical records. Before VCTE, the patient fasted for a minimum of 4 hours and remained alcohol-free for 7 days. VCTE was the first imaging test performed using the FibroScan Compact 530 (Echosens, Paris, France). Each patient was placed in the supine position with the right arm in abduction and the ipsilateral hand resting under the head. First, a US examination was conducted to assess liver size, echogenicity, surface irregularity, exclusion of masses, presence of ascites, presence or absence of hepatic vein wall, and spleen size; in addition, Doppler examination of both the portal and splanchnic circulation was performed. Afterward, upon breath-hold at the end of expiration, 10 measurements were obtained with the M-probe placed in the area of the right hepatic lobe (RHL) through an intercostal space, and a median value was given by the equipment. The transition to an XL probe was based on a prompt from the VCTE automatic probe selection tool. According to the median value, liver fibrosis was staging as 3 to 4 (F3–4) according to standard VCTE cutoff values: ≥9.5 kPa for NAFLD, hepatitis B virus (HBV) and C virus (HCV) infection, ≥10.5 kPa for primary biliary cholangitis (PBC) and autoimmune hepatitis, ≥11.5 kPa for HIV/HCV coinfection, and ≥12 kPa for alcohol-related cirrhosis. The NAFLD cutoff value of ≥9.5 kPa was also considered for drug-induced^[9]^ and cryptogenic cirrhosis.

#### EUS–guided 2-dimensional shear wave elastography

EUS-SWE was performed by R.V. and F.D.B., who were blinded to the clinical records and VCTE findings. Both groups underwent VCTE and EUS-SWE. SW measurement was performed with the Arrieta 850 EUS console (Fujifilm, Tokyo, Japan) using a linear ultrasound video gastroscope EUS-J10 (Pentax Medical, Hoya Corp, Japan). Under general anesthesia, patients underwent EUS-SWE following the World Federation for Ultrasound in Medicine and Biology (WFUMB) guidelines.^[3]^ The patient was lying in the supine position. The transducer was positioned in the gastric window to visualize right liver segment number 5 and left liver segment 2 or 3, with the capsule parallel to the transducer.^[10]^ The elastogram region of interest (ROI) was placed within the liver tissue at a distance ≥10 mm beneath the hepatic capsule in an area free of vessels and artifacts. A 10-mm circular ROI was placed within the elastogram at a depth of 4–5 cm from the skin, and a minimum of 10 successful kPa measurements were obtained, providing information about the presence or absence of liver stiffness. Each EUS-SWE took approximately 40 minutes. After EUS-SWE, a second physician (J.B.-B.) opened the label to confirm the patient's group. The patient continued to undergo EUS-guided 2-lobe biopsy in both study groups as follows: first, the left liver lobe was punctured from the gastric subcardial region. Then, puncture of the right liver lobe was performed from the gastric antrum or duodenal bulb. After an accurate inspection of the interposing vessels between the EUS probe and the puncture site, a 19G FNA needle was inserted into the liver. Five passes were performed without aspiration and 5 using half-open aspiration syringe. A single puncture was performed if the macroscopic onsite examination of the tissue fragment was considered appropriate.

### Data extraction, integration, and study endpoint

The initial clinical assessment data, VCTE parameters, EUS-SWE parameters, and EUS-guided 2-lobe liver biopsy results were retrieved through a data collection process designed specifically for each stage of the study. Finally, the data were integrated into a single database and encrypted into an MS Access-compatible database. The study endpoint was fibrosis estimated by VCTE, EUS-SWE, and liver biopsy. For VCTE, a F3–4 was considered to indicate advanced fibrosis or cirrhosis. A liver biopsy indicated cirrhosis at a Kleiner's F4 score^[11]^ and Meta-analysis of Histological Data in Viral Hepatitis (METAVIR) F4 score in case of HBV^[12]^ and HCV infection.^[13]^ Steatosis was assessed using the Brunt's score. For EUS-SWE, the cutoff value for estimating cirrhosis is explained in the Statistical Analysis section.

### Statistical analysis

#### Technical considerations

Data were analyzed by a certified gastroenterologist and biostatistician (M.P.-T.) using R v.4.0 (R Foundation for Statistical Computing; Vienna, Austria). A *P* value <0.05 was considered to indicate statistical significance.

#### Sample size

The sample size was based on the proportion of cases with a high liver stiffness measurement (LSM) compatible with cirrhosis based on an EUS strain histogram (SH) among cirrhosis cases (87.5%) *versus* controls (31%).^[14]^ Using a 2-tailed formula, a case *versus* controls ratio of 1, and alpha and beta errors of 5% and 20%, respectively, a sample size of not less than 25 individuals per study group was estimated with 80% power.

#### Descriptive analysis

Numeric variables with a Gaussian distribution are presented as the mean (standard deviation, SD). Data with a skewed distribution are presented as the median (interquartile range [IQR]). Categorical variables are presented as the frequency (95% confidence interval [CI]).

#### Inferential analysis

Baseline data were compared among both study groups using hypothesis testing. The association and concordance between VCTE and EUS-SWE were evaluated with Spearman's rank correlation (rho) and the intraclass correlation coefficient (ICC), and the results are presented as a box plot and a Bland-Altman plot, respectively. The EUS-SWE cutoff for liver cirrhosis diagnosis was estimated through Youden's index, considering EUS-guided liver biopsy as the gold standard. The following measures of the overall accuracy of VCTE and EUS-SWE for liver cirrhosis diagnosis were calculated: sensitivity, specificity, positive predictive value (PPV), negative predictive value (NPV), and observed agreement. The corresponding area under the receiver operating characteristic curve (AUROC) was also calculated.

## RESULTS

### Descriptive analysis

From March 2020 to November 2021, 59 patients underwent VCTE and EUS-SWE evaluations. Of these 59 patients, 29 had a diagnosis of cirrhosis confirmed by EUS-guided 2-lobe liver biopsy in agreement with Kleiner's score, whereas 30 were controls [Table 1]. The mean age was 63.5 years (IQR 56.6–70.5), and 42 were female (71.1%). The median BMI was 27.3 kg/m^2^ in the cirrhosis group and 25.4 kg/m^2^ in controls (*P* = 0.1998). The median laboratory results for the cirrhosis group were 109 × 10^9^/L platelet count, 75 U/L AST, and 60 U/L ALT, whereas the corresponding values in controls were 239 × 10^9^/L (*P* < 0.001), 32.8 U/L (*P* < 0.001), and 35 U/L (*P* = 0.005). During VCTE, the controlled attenuation parameter (CAP) score was 229 dB/m in the cirrhosis group and 221 dB/m in controls (*P* = 0.6061).

**Table 1:** Baseline characteristics of the study group.

	Cirrhosis (*n* = 29)	Control (*n* = 30)	*P*
Age (yr), median (IQR)	65 (61–73)	62 (51.5–66)	0.0731*^a^*
Adults (40–64), *n* (%)	15 (51.7)	11 (36.7)	
Elderly (≥65), *n* (%)	14 (48.3)	13 (43.3)	
Sex (female), *n* (%)	20 (69.0)	22 (73.3)	0.9340*^b^*
BMI (kg/m^2^), median (IQR)	27.3 (24.2–35.2)	25.4 (23.4–29.4)	0.1998*^a^*
Underweight (<18.5), *n* (%)	—	3 (10.0)	
Normal weight (18.5–24.9), *n* (%)	10 (34.5)	12 (40.0)	
Overweight (25–29.9), *n* (%)	7 (24.1)	8 (26.7)	
Obese (≥30), *n* (%)	12 (41.4)	7 (23.3)	
Platelet count (×10^9^/L), median (IQR)	109 (88–131)	239 (213–270)	<0.001*^a^*
50–150, *n* (%)	25 (86.2)	2 (6.7)	
≥150, *n* (%)	4 (13.8)	28 (93.3)	
AST (U/L), median (IQR)	75.0 (44.1–114)	32.8 (26.2–46.5)	<0.001*^a^*
≤40, *n* (%)	5 (17.2)	18 (60.0)	
>1 to 3 times ULN, *n* (%)	18 (62.1)	10 (33.3)	
>3 to 5 times ULN, *n* (%)	1 (3.4)	1 (3.3)	
>5 to 10 times ULN, *n* (%)	5 (17.2)	1 (3.3)	
ALT (U/L), median (IQR)	60.0 (37.0–105)	35.0 (30.0–54.0)	0.0055*^a^*
≤45, *n* (%)	13 (44.8)	19 (63.3)	
>1 to 3 times ULN, *n* (%)	13 (44.8)	11 (36.7)	
>3 to 5 times ULN, *n* (%)	2 (6.9)	2 (6.7)	
>5 to 10 times ULN, *n* (%)	3 (10.3)	—	
Steatosis (S) estimated by CAP (dB/m), median (IQR)	229 (181–301)	221 (160–301)	0.6061*^a^*
S0 (<302), *n* (%)	22 (75.9)	24 (80.0)	
S1 (302–331), *n* (%)	3 (10.3)	3 (10.0)	
S2 (332–337), *n* (%)	—	1 (3.3)	
S3 (>337)	4 (13.8)	2 (6.7)	
CAP IQR, median (IQR)	33.0 (19.5–52.0)	36.0 (30.0–56.0)	0.0397*^a^*
Cause of cirrhosis, *n* (%)			n/c
NAFLD	17 (58.6)	—	
Alcohol	9 (31.0)	—	
Drug-induced	1 (3.4)	—	
Cryptogenic	2 (6.9)	—	
Alcohol intake among patients with alcohol-related cirrhosis (*n* = 9) (g/wk), *n* (%)			n/c
≈ 80	5 (55.6)	—	
>80	4 (44.4)	—	
Child-Pugh score, *n* (%)			n/c
A	21 (72.4)	—	
B	8 (27.6)	—	
MELD score, median (IQR)	8.47 (6.65–10.2)	—	n/c
0–9	16 (55.2)	—	
10–19	13 (44.8)	—	
Presence of varices, *n* (%)			<.001^c^
No varix	9 (31.0)	30 (100.0)	
Gastric varices	9 (31.0)	—	
Esophageal varices	11 (37.9)	—	

ALT, alanine aminotransferase; AST, aspartate aminotransferase; BMI, body mass index; CAP, controlled attenuation parameter; dB/m, decibels per meter; IQR, interquartile range; MELD, Model for End-Stage Liver Disease; NAFLD, nonalcoholic fatty liver disease; ULN, upper limit of the normal range; n/c, not calculable.

*^a^* Mann-Whitney *U* test.

*^b^* Pearson's chi-squared test with Yates' continuity correction.

*^c^* Fisher's exact test for count data.

The causes of cirrhosis were NAFLD in 17/29 (58.6%), alcohol consumption in 9/29 (31%), drug-induced in 1/29 (3.4%), and cryptogenic in 2/29 (6.9%). A Child-Pugh A score was determined in 21/29 cirrhosis patients, whereas a Child-Pugh B score was determined in 8/29. The median MELD score in the cirrhosis group was 8.47. Among the patients with cirrhosis, 9/29 had no varix (31.0%), 9/29 had gastric varices (31.0%), and 11/29 had esophageal varices (37.9%), data helpful to determine the extent of the severity at the time of diagnosis. In the cirrhosis group, the median liver biopsy length was 18 mm (IQR 16–22) with no statistical difference between both hepatic lobes. The baseline patient characteristics are summarized in Table 1, and the histopathological profile of liver biopsies in Supplementary Table 1, http://links.lww.com/ENUS/A368.

### VCTE and EUS-SWE measurements

**Figure 1:**
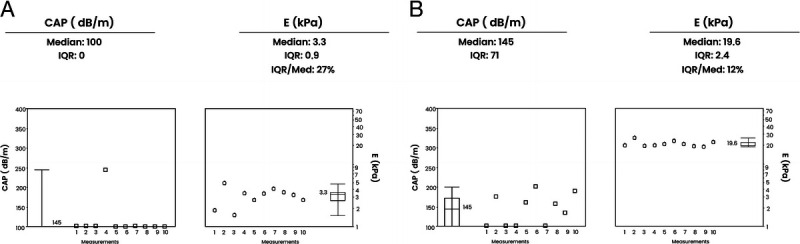
Vibration-controlled transient elastography evaluation. A, Case no. 55. A 63-year-old female with chronic dyspepsia and abdominal pain, a past medical history of rheumatoid arthritis, current methotrexate therapy, and a family history of pancreatic cancer. This participant underwent vibration-controlled transient elastography (VCTE) to rule out hepatotoxicity, and the calculated VCTE was 3.3 kPa. B, Case no. 27. An 82-year-old male with a 36-month history of cirrhosis and previous alcohol consumption of 150 g/w underwent VCTE to rule out complications such as esophageal varices and hepatocellular carcinoma, and the calculated VCTE was 19.6 kPa.

The median LSM estimated by VCTE was 17.8 kPa in the cirrhosis group (IQR 11.8–26.8) and 4.6 kPa in the control group (3.7–5.3; *P* < 0.001) [Figure 1]. A total of 24/29 participants in the cirrhosis group had a VCTE of F3–4 (82.8%), whereas 30/30 of those in the control group had a score of F0–2 (100%). The dispersion of LSM by VCTE did not significantly differ between study groups: 17.4 in cirrhosis patients and 16.5 in controls (*P* = 0.75) [Table 2].

**Table 2:** Liver stiffness measurement through vibration-controlled transient elastography (VCTE) and EUS–guided 2-dimensional shear wave elastography (EUS-SWE) for the cirrhosis and control groups.

	Cirrhosis (*n* = 29)	Control (*n* = 30)	*P*
Vibration-controlled transient elastography (VCTE)			
Median fibrosis (F) estimated by VCTE (kPa)	17.8 (11.8–26.8)	4.6 (3.7–5.3)	<0.001*^a^*
F0–2, *n* (%)	5 (17.2)	30 (100.0)	
F3–4	24 (82.8)	—	
VCTE IQR	2.5 (1.6–3.9)	0.7 (0.4–0.9)	<0.001*^a^*
VCTE IQR/median (dispersion)	17.4 (11.7–20.9)	16.5 (10.8–20.8)	0.7559*^a^*
EUS–guided 2-dimensional shear wave elastography (EUS-SWE)			
RHL-EUS-SWE median	27.0 (24.1–31.4)	5.6 (4.6–7.2)	<0.001*^a^*
≤10.7, *n* (%)	1 (3.4)	27 (90.0)	
>10.7	28 (96.6)	3 (10.0)	
RHL-EUS-SWE IQR	5.4 (3.2–7.8)	1.9 (0.9–2.7)	<0.001*^a^*
RHL-EUS-SWE IQR/median (dispersion)	20.5 (13.3–29.6)	29.4 (17.8–40.2)	0.1585*^a^*
LHL-EUS-SWE median	25.0 (19.3–31.3)	6.45 (4.43–8.10)	<0.001*^a^*
≤14.0, *n* (%)	1 (3.4)	29 (96.7)	
>14.0	28 (96.6)	1 (3.3)	
LHL-EUS-SWE IQR	6.20 (4.40–8.25)	2.33 (1.40–3.23)	<0.001*^a^*
LHL-EUS-SWE IQR/median (dispersion)	23.9 (17.9–28.7)	36.7 (21.4–58.3)	0.0234*^a^*

Continuous variables are described as the median (interquartile range [IQR]).

kPa, kilopascal; LHL, left hepatic lobe; RHL, right hepatic lobe, VCTE, vibration-controlled transient elastography.

*^a^* Mann-Whitney *U* test.

**Figure 2:**
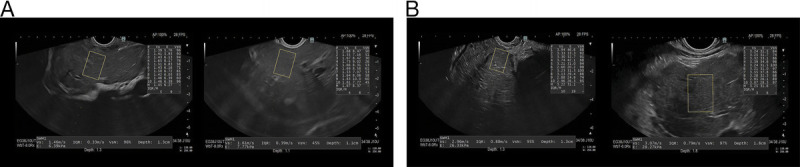
EUS–guided 2-dimensional shear wave elastography (EUS-SWE) evaluation. A, Case no. 37. A 63-year-old female with chronic abdominal bloating, previous US results of a normal liver and a gallbladder polyp, and a previous EUS result of minimal inflammatory pancreatic changes. She underwent EUS-SWE, which revealed an LSM of 6.27 kPa in the RHL and 8.37 kPa in the LHL. B, Case no. 09. A 49-year-old female with a 10-day history of jaundice and epigastric pain radiating throughout the abdomen and recent weight loss of 24 lb. She has a past medical history of cirrhosis and diabetes. EUS-SWE revealed an LSM of 31.1 kPa in the RHL and 31.6 kPa in the LHL. LHL, left hepatic lobe; SWE, shear wave elastography; LSM, liver stiffness measurement.

EUS-SWE evaluation of the RHL showed a median LSM of 27 kPa in the cirrhosis group and 5.6 kPa in controls (*P* < 0.01), using a cutoff value of 10.7 kPa obtained by Youden's index [Figure 2]. A total of 28/29 and 3/30 patients had an LSM value >10.7 kPa in the cirrhosis and control groups, respectively [Table 2]. As in VCTE, dispersion of LSM by RHL-EUS-SWE was not significantly different between study groups: 20.5 in the cirrhosis group and 29.4 in controls (*P* = 0.15).

Left hepatic lobe (LHL) EUS-SWE assessments revealed a median LSM of 25 and 6.45 kPa in the cirrhosis and control group, respectively. A cutoff value of 14 kPa was obtained; 28/29 of participants in the cirrhosis group and 1/30 of those in controls had an LSM value >14 kPa (*P* < 0.01). In opposition to LSM dispersion throughout VCTE and RHL-EUS-SWE measurements, the dispersion of LSM by LHL-EUS-SWE were 23.9 and 36.7 in the cirrhosis and control groups, respectively (*P* = 0.02) [Table 2].

In general, there was a very good association and concordance between VCTE-measured LSM and EUS-SWE. The association between VCTE and EUS-SWE was rho = 0.758 (*P* < 0.001) in the RHL. Supplementary Figure 1A, http://links.lww.com/ENUS/A369, illustrates case distribution by RHL-EUS-SWE measurement >10.7 kPa, represented by a red dotted line. Only in 1/29 non-variceal cirrhosis patients (light blue points), RHL-EUS-SWE was ≤10.7 kPa; 3/30 controls (dark blue points) reached an RHL-EUS-SWE >10.7 kPa. The association between VCTE and EUS-SWE was rho = 0.764 (*P* < 0.001) in the LHL. Supplementary Figure 1B, http://links.lww.com/ENUS/A369, illustrates case distribution by LHL-EUS-SWE measurement >14.0 kPa (red dotted line). Only in 1/29 esophageal-variceal cirrhosis patients (red points), LHL-EUS-SWE was <14.0 kPa; 1/30 controls reached an LHL-EUS-SWE >14.0 kPa. In 10/29 cirrhosis patients, VCTE determined F0–2: 2/10 non-variceal (light blue points) and 3/10 gastric varices (orange points), but there was not any control with VCTE F3–4. Meanwhile, concordance between VCTE and EUS-SWE was ICC = 0.641 (95% CI, 0.474–0.763; *P* < 0.001) in the RHL [Supplementary Figure 1C, http://links.lww.com/ENUS/A369] and ICC = 0.548 (95% CI, 0.358–0.694; *P* < 0.001) in the LHL [Supplementary Figure 1D, http://links.lww.com/ENUS/A369]. Cases with esophageal varices seem to present higher median fibrosis in comparison with gastric-variceal cases when measured by VCTE (23.3 *vs.* 11.8 kPa; *P* = 0.0175) and EUS-SWE in LHL (31.3 *vs.* 22.8 kPa; *P* = 0.0404). Regarding RHL, the difference in fibrosis among esophageal *versus* gastric-variceal cases was not significant [Supplementary Table 2, http://links.lww.com/ENUS/A370].

### VCTE and EUS-SWE diagnostic accuracy

**Figure 3:**
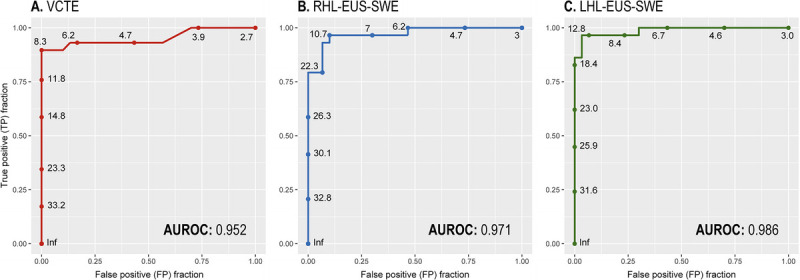
Area under the receiver operating characteristic curve (AUROC) for diagnosing cirrhosis. A, Vibration-controlled transient elastography (VCTE). B, EUS–guided 2-dimensional shear wave elastography (EUS-SWE) stiffness measurement of the right hepatic lobe (RHL). C, EUS-SWE stiffness measurement of the left hepatic lobe (LHL).

The sensitivity, specificity, PPV, NPV, and observed agreement for LSM by VCTE were 82.8%, 100%, 100%, 85.7%, and 91.5%, respectively [Table 3]. In contrast, RHL- and LHL-EUS-SWE had 96.6% and 96.6% sensitivity, 90% and 96.7% specificity, 90.3% and 96.6% PPV, 96.4% and 96.7% NPV, and 93.2% and 96.6% observed agreement, respectively. The AUROCs for predicting liver fibrosis were as follows: VCTE, 0.95; RHL-EUS-SWE, 0.97; and LHL-EUS-SWE, 0.98 [Figure 3].

**Table 3:** Overall diagnostic accuracy (*n*/T; % [95% CI]) for diagnosing cirrhosis based on stiffness measurements obtained by vibration-controlled transient elastography (VCTE) *versus* EUS–guided 2-dimensional shear wave elastography (EUS-SWE) in both the right and left hepatic lobes (RHL and LHL, respectively).

	VCTE ≥12*^a^* or ≥9.5^b^ kPa	RHL-EUS-SWE >10.7 kPa	LHL-EUS-SWE >14.0 kPa
Sensitivity	24/29; 82.76 (64.23–94.15)	28/29; 96.6 (82.2–99.9)	28/29; 96.6 (82.2–99.9)
Specificity	30/30; 100 (88.43–100)	27/30; 90 (73.5–97.9)	29/30; 96.7 (82.8–99.9)
PPV	24/24; 100 (85.75–100)	28/31; 90.3 (74.2–97.9)	28/29; 96.6 (82.2–99.9)
NPV	30/35; 85.71 (69.74–95.19)	27/28; 96.4 (81.7–99.9)	29/30; 96.7 (82.8–99.9)
Observed agreement	54/59; 91.53 (81.32–97.19)	55/59; 93.2 (83.5–98.1)	57/59; 96.6 (88.3–99.6)
AUROC	0.952	0.971 (*P* = 0.3881*^c^*)	0.986 (*P* = 0.2906*^d^*)

*^a^* Cutoff value for alcohol-related cirrhosis.

*^b^* Cutoff value for nonalcoholic fatty liver disease (NAFLD), drug-induced cirrhosis, or cryptogenic cases.

*^c^* DeLong's test for correlation of AUROC between VCTE and RHL-EUS-SWE.

*^d^* DeLong's test for correlation of AUROC between VCTE and LHL-EUS-SWE.

CI, confidence interval; kPa, kilopascal; NPV, negative predictive value; PPV, positive predictive value; AUROC, area under the receiver operating characteristic.

## DISCUSSION

In this study, we compared VCTE and EUS-SWE for the diagnosis of liver cirrhosis. VCTE had a lower sensitivity (82.8%) than EUS-SWE (96.6%) for the diagnosis of cirrhosis. The median LSM was 18.8 kPa by VCTE, 27 kPa by RHL-EUS-SWE, and 25 kPa by LHL-EUS-SWE. We defined cirrhosis cutoff values of 10.7 and 14 kPa for RHL-EUS-SWE and LHL-EUS-SWE, respectively. Using VCTE standard cutoff values, it was less sensitive (65.5%) than SWE (96.6%) but had a higher specificity (100%) when compared with RHL-EUS-SWE (90%) and LHL-EUS-SWE (96.7%). All procedures had an accuracy above 0.95.

Using the reliable criteria of IQR/median <30%, we used the standard VCTE cutoff value for F3–4, ≥12 and ≥9.5 kPa, for alcohol-related cirrhosis, and NAFLD, drug-induced and cryptogenic cirrhosis, respectively. A meta-analysis from 2022 that included 37 primary studies analyzed the diagnostic utility of VCTE in patients with cirrhosis and defined cutoff values of 8–10 kPa based on previous literature.^[15]^ However, a recent original study using Boursier's criteria concluded that the optimal cutoff value for LSM by VCTE to detect cirrhosis is 10.3 kPa.^[16]^

Similar to EUS-SWE, acoustic radiation force impulse imaging (ARFI) allows a noninvasively liver fibrosis assessment but through a transparietal 2D-SWE US. In a previous multicenter international cohort study that aimed to stratify the 2-year mortality risk of cirrhotic patients, the optimal cutoff value by 2D-SWE-US was determined to be 20 kPa in compensated patients (maximum MELD score of 10 points) based on Youden's index.^[17]^ A cross-sectional study utilizing the Baveno VI criteria reported a median LSM of 9.9 kPa by 2D-SWE-US.^[3]^

The recent American study addressed by Kohli et al.^[18]^ is the first publication that established an EUS-SWE cutoff value of 18.5 and 24 kPa to identify cirrhosis in the RHL and LHL from individuals with NAFLD, respectively. In our study, using the same Youden's index calculation, we determined cutoff values of 10.7 and 14 kPa for the RHL and LHL, respectively. Differences between cutoff values from both studies can be explained by a clinical fact. Our study was compounded by 19/59 patients with obesity (32.2%), a control group with median steatosis (S) estimated by the controlled attenuated parameter (CAP; dB/m) of 221 dB/m (160–301), and a cirrhosis group compounded by 17/29 NAFLD (58.6%) and only 7/29 S1–3 cases (24.1%). Kohli et al. analyzed a sample of 42 patients with NAFLD, 28/42 patients with obesity (66.7%), and a mean CAP of 290.4 ± 95.8 dB/m. Meanwhile, both studies concluded with an LSM difference between 3.3 and 5.5 kPa, showing always the LHL with the highest cutoff value. This difference can be explained by a technical fact. The hepatic lobe may be influenced by angulation of the echoendoscope and diaphragmatic movement, which may impact the propagation of the shear wave. This angulation is lower when assessing the RHL, but higher when the LHL is assessed through the gastric window.^[18]^

A previous meta-analysis reported a sensitivity of 84% and a specificity of 78% using cutoff values of 8–10 kPa for LSM with VCTE.^[16]^ Additionally, a multiple threshold meta-analysis including 1278 total subjects determined a sensitivity of 80% and a specificity of 77% for VCTE at a cutoff value of 8.9 kPa, whereas a cutoff value of 9.5 kPa achieved 76% sensitivity and 80% specificity.^[19]^ Another previous study established that using a cutoff value of 16.1 kPa for LSM with SWE achieves a sensitivity of 78% and a specificity of 72%.^[20]^ However, in our study, the standard VCTE cutoff values and the EUS-SWE values of 10.7 and 14 kPa yielded sensitivities of 82.8%, 96.6%, and 96.6% and specificities of 100%, 90%, and 96.7% for VCTE, RHL-EUS-SWE, and LHL-EUS-SWE, respectively.

A cross-sectional study found 87.9% agreement between SWE and VCTE regarding a diagnosis of cirrhosis.^[3]^ Ayonrinde et al. reported an AUROC of 0.8 for VCTE versus 0.84 for SWE (*P* = 0.21). Shima et al.^[15]^ stated that using the criterion IQR/median <30% recommended by the European Federation of Societies for Ultrasound in Medicine and Biology (EFSUMB) and the American Gastroenterological Association (AGA), VCTE had an AUROC of 0.91–0.94 Moreover, the reported AUROC values from a prospective study were 0.90 for VCTE, 0.90 for RHL-EUS-SWE, and 0.96 for LHL-EUS-SWE.^[18]^ Our study had similar results compared to the last 2 studies, with similar AUROC values for VCTE, RHL-EUS-SWE, and LHL-EUS-SWE.

Recently, we have demonstrated that implementation of EUS with elastography of the liver has diagnostic value for portal hypertension secondary to chronic liver disease, not only offering endosonographic but elastographic evaluation in a single diagnostic test surpassing the limitations VCTE might face (*i.e*., obese patients and presence of ascites, among others).^[14]^ Furthermore, EUS-SWE has many benefits over VCTE. One of them is the ability to evaluate both liver lobes with the theoretical benefit of overcoming the heterogeneous distribution of fibrosis or cirrhosis and therefore overcoming discordance among liver biopsies. In addition, EUS is not affected by abdominal wall interference in patients with ascites or obesity. Moreover, during EUS-guided evaluations, endosonographers can investigate esophageal and gastric varices and, if indicated, perform a EUS-guided liver biopsy in the same endoscopic session, overcoming the need for repetitive evaluations. However, this approach is not yet a standard recommendation due to its low availability, high-cost procedure and accessories compared to percutaneous approach, and the challenges caused by the low number of EUS training facilities worldwide.

To the best of our knowledge, this is the first study that compares VCTE *versus* EUS-SWE for cirrhosis assessment in Hispanics. We provide novel cutoff values for the diagnosis of cirrhosis using EUS-SWE. As we said, they are similar to an American study: 18.4 and 24 kPa for cirrhosis in the LHL and RHL, whereas ours are 10.7 and 14 kPa, respectively.^[18]^ It demonstrates the feasibility of this technique as an alternative tool in the diagnosis workup of cirrhosis. Moreover, to evaluate the liver parenchyma through endosonography and elastography, a direct acquisition of tissue for histological evaluation in a single procedure is also possible. It plays a relevant role mainly in patients with doubt diagnosis or confirm cirrhosis in cryptogenic cases.

Our study has some limitations. Its sample size was powered to differentiate cirrhosis patients from controls, but not for grading liver fibrosis, or for subanalyzing between different cirrhosis etiologies. Both imply a larger sample or different studies. It is important to highlight that our study but also the one addressed by Kohli et al.,^[18]^ both designed in late 2020, used the traditional NAFLD definition, instead of the current and more correct concept of metabolic dysfunction-associated steatotic liver disease (MASLD), proposed in 2023.^[21]^ Although a higher accuracy was achieved with EUS-SWE than with VCTE, cutoff values for LSM were calculated using Youden's index based on data with certain dispersion, especially in LHL. This may depend on the experience of a single-center study.

In conclusion, EUS-SWE and VCTE are reliable diagnostic methods for measuring LSM in patients with cirrhosis, without superiority over each other demonstrated by the AUROC. Moreover, the observed agreement was significantly higher for LHL-EUS-SWE than for RHL-EUS-SWE or VCTE, indicating that EUS-SWE is a feasible alternative when VCTE seems unreliable (i.e., patients with ascites or obesity). This research must continue in a larger, multicenter trial that pursues to grade liver fibrosis through EUS-SWE, considering specific liver fibrosis. To be consistent with the current literature, it is advisable to use the new concept of MASLD in further research.

## Acknowledgments

None.

## Source of Funding

None.

## Ethical Approval

The local institutional review board approved the study, which was conducted following the Declaration of Helsinki.

## Informed Consent

All the enrolled patients signed informed consent forms before participating in the study.

## Conflict of Interest

Carlos Robles-Medranda is a key opinion leader and consultant for Pentax Medical, Steris, Micro-tech, G-Tech Medical Supply, CREO Medical, EndoSound, and mdconsgroup. The other authors have nothing to disclose.

## Author Contributions

Domenica Cunto, Miguel Puga-Tejada, and Roberto Oleas performed acquisition of data, analysis and interpretation of data, critical revision of the manuscript for important intellectual content, and final approval of the version to be published. Maria Egas-Izquierdo, Martha Arevalo-Mora, and Jorge Baquerizo-Burgos contributed to the acquisition of data, critical revision of the manuscript for important intellectual content, and final approval of the article. Raquel del Valle R, Juan Alcivar-Vasquez, Fernanda Dal Bello, and Hannah Pitanga-Lukashok contributed to the conception and design of the work, critical revision of the manuscript for important intellectual content, and final approval of the version to be published. Carlos Robles-Medranda performed the study conception, project management, acquisition of data, critical revision of the manuscript for relevant intellectual content, and final approval of the article.

## Data Availability Statement

The database of the study is not publicly available cause of local regulations based on the Ecuadorian Organic Law on the Protection of Personal Data.

## Clinical Registry

Clinical Trial No. NCT04644055.
